# An Experimental Investigation of the Effects of Block Proportion on Bimrocks, Considering Different Block-to-Matrix Strength Ratios

**DOI:** 10.3390/ma17051114

**Published:** 2024-02-28

**Authors:** Yanran Hu, Shaorui Sun, Yuyong Sun, Jihong Wei, Huilin Le, Kai Li, Bohan Zhao

**Affiliations:** 1 School of Earth Sciences and Engineering, Hohai University, Nanjing 211111, China; hyr@hhu.edu.cn (Y.H.); wjhfish@hhu.edu.cn (J.W.); lehuilin@hhu.edu.cn (H.L.); llkk@hhu.edu.cn (K.L.); 221309080031@hhu.edu.cn (B.Z.); 2 School of Architectural Engineering, Tongling University, Tongling 244000, China; sunyuyon2@126.com

**Keywords:** bimrocks, volumetric block proportion, block-to-matrix strength ratio, experimental investigation

## Abstract

The rock block proportion is one of the most important factors affecting the mechanical properties of bimrocks. Under different block-to-matrix strength ratios, the influence of rock block proportion is different. To explore the influence of rock block proportion on the mechanical properties of specimens under different block-to-matrix strength ratios, a new indoor test method for making bimrocks was proposed. A uniaxial compression test and a direct shear test were carried out on specimens with different rock block proportions. The results show that this method can control the block-to-matrix strength ratio well, and the influence of rock block proportion is obviously different under different block-to-matrix strength ratios. The strong matrix sample will decrease significantly after reaching the peak compressive strength, while the weak matrix will decrease slowly after reaching the peak strength. The rock block proportion is negatively correlated with the uniaxial compressive strength of strong matrix samples (the reduction was 12.53%) and is positively correlated with the uniaxial compressive strength of weak matrix samples as a whole, but it changes when block proportion is more than 50%. With the increase in normal stress and rock block proportion increases from 30% to 60%, the shear failure zone of the weak matrix sample increases, and the cracks are inclined, while the strong matrix sample has more secondary cracks. The results of this study also show that the effect of volumetric block proportion (VBP) on the internal friction angle and cohesion of the sample is less related to the block-to-matrix strength ratio.

## 1. Introduction

Bimrocks are complex geological materials composed of rock blocks and matrices with different mechanical properties, and have obvious inhomogeneity and anisotropy. These materials such as mélange [1,2], fault breccia [3,4], conglomerate [5], and lithified colluvium [6] are inevitable to encounter in engineering. Therefore, it is necessary to understand their strength characteristics and failure behaviors. However, due to many influencing factors such as rock block proportion [7,8], rock block size [9,10], rock block shape [11,12], matrix strength [13], matrix rock strength ratio [14], and the block–matrix interface [15], the research of bimrocks has always been a hot and difficult problem.

Rock block proportion (RBP) is one of the most important factors affecting the mechanical properties of bimrocks, and it can be divided into volumetric block proportion (VBP) [16,17] and weight block proportion (WBP) [18,19,20]. According to different classification in two-dimensional studies, there is also the term ABP (areal block proportions) [21]. Among the regular cylindrical or square specimens, VBP is more widely used because the calculation of the volume ratio is more accurate and convenient. However, considering the void inside the specimen, it is difficult to make high VBP bimrock specimens indoors. Therefore, WBP is used in the case of high block proportion [22,23,24]. Before considering the influence of block proportion, it is necessary to have a certain understanding of the strength relationship between block and matrix, because the influence of RBP is different or even opposite in the case of different matrices and block strengths. When the strength of the rock block is much higher than that of the matrix, and the block proportion is less than 70%, the uniaxial compressive strength of the sample shows an increasing trend with the increase in the block proportion [25,26,27], but when the block proportion exceeds 70%, the uniaxial compressive strength of the sample shows a decreasing trend [22]. Kahraman and Alber [28] found that the uniaxial compressive strength of the sample showed a decreasing trend with the increase in VBP, when studying a fault breccia with weak blocks and a strong matrix; the uniaxial compressive strength of the sample is basically the same when the VBP exceeds 70%.

Previous studies on the influencing factors of rock block proportion in bimrocks are limited to the level of rock strength above (or below) matrix strength. There are few studies in the literature that comprehensively consider the two situations. The measurement of rock strength in bimrocks samples has always been a difficult problem, especially through laboratory tests. In this study, the relative relationship between the strength of the block and the matrix is well controlled by the artificial block material, whose uniaxial compressive strength and shear strength are greater than (or less than) the matrix in the laboratory. Through the indoor uniaxial compression test and direct shear test, the strength characteristics and failure characteristics of the samples with different VBPs under different matrix block strength ratios are analyzed. The research results solved the problem of how to quantitatively characterize the strength ratio of block and matrix and studied the influence of VBP change on the mechanical properties of samples in two cases, providing an important reference for the related research on the strength ratio of bimrocks’ matrices and blocks.

## 2. Materials and Methods

### 2.1. Obtaining Blocks and Sample Preparation

Cement and quartz sand are common materials for making artificial bimrocks. By controlling the ratio of cement and quartz sand, a variety of pure matrix samples with different strengths can be obtained. The bimrock’s type is the weak matrix type when the strength of the block is much greater than the matrix strength, and the strong matrix type when the strength of the block is much smaller than the matrix strength. The cement used in the test is PO 42.5 cement, quartz sand is mainly obtained from quartz crushing, and the particle size is 120 mesh. According to the pre-test results, the production ratio of the rock block is selected as cement and quartz sand 1:1, the production ratio of a weak matrix is 1:3, and the production ratio of a strong matrix is 3:1. Before making the rock block, it is necessary to carry out a mechanical test on the sample under the ratio. After obtaining the strength parameters of the sample, such as uniaxial compressive strength, shear strength, cohesion, and internal friction angle, the production of the rock block is started.

**Figure 1:**
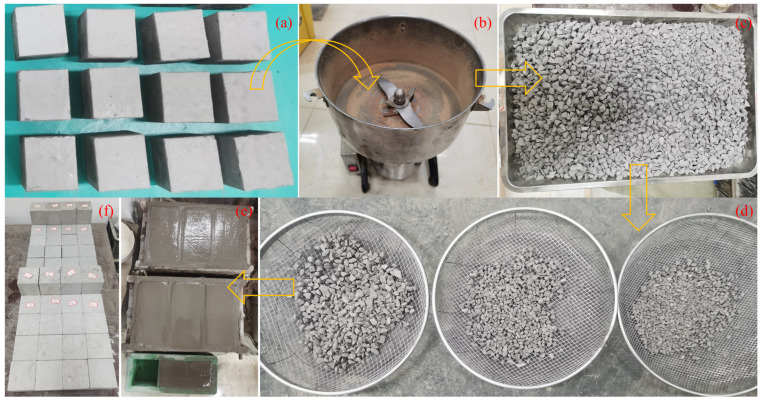
The preparation of samples. (**a**) The raw materials of rock block, (**b**) crusher, (**c**) crushed rock blocks, (**d**) rock blocks screening, (**e**) sample before demolding, and (**f**) sample after curing.

The production of the rock block mainly includes two steps: fragmentation and screening. First, the prepared sample was preliminarily crushed with a hammer and then the fragments were placed in a crusher for further crushing (Figure 1a,b). The crushed rocks (Figure 1c) were screened multiple times (Figure 1d) and the rocks with a particle size of 6~12 mm were selected as test supplies. Four kinds of VBP, 30%, 40%, 50%, and 60%, were set up in the test. The corresponding quantity of rock block, cement, and quartz sand were first stirred, and then 0.2 times the total mass of cement and quartz sand dry powder was added. Finally, the sample can be obtained by standing it in the corresponding mold for one day (Figure 1e), and the relevant mechanical test can be carried out after curing at room temperature for 28 days (Figure 1f). The test specimens are shown in Figure 1e, including cuboid specimens with a size of 70 mm × 70 mm × 140 mm and cube specimens with a size of 70 mm × 70 mm × 70 mm, which are used for uniaxial compression tests and direct shear tests, respectively.

The total number of samples was 110, which were divided into the same two groups with 55 samples in each group to ensure the accuracy of the test results. In each group, 11 uniaxial compression samples and 44 direct shear samples were included.

### 2.2. Equipment and Tests

**Figure 2:**
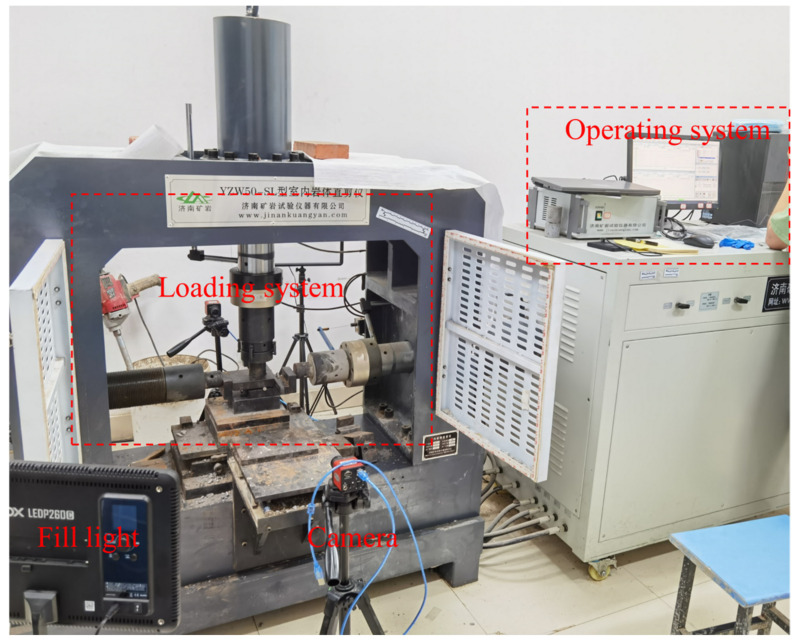
Testing equipment.

The YZW50-SL equipment was used in the tests (Figure 2) (Hohai University, Nanjing, China), the pressure threshold of the equipment is 500 kN. The displacement in the compression and shear tests can be measured automatically. The equipment mainly includes the loading system and the operating system, the loading method adopts displacement control in the uniaxial compression test and the direct shear test. The loading speed is constant at 0.1 mm/min in the uniaxial compression test, while the stress–strain curve drops sharply, and the test is then manually terminated. The termination condition of the shear test is when the displacement reaches 7 mm or the curve drops significantly. The shear rate is 0.05 mm/min. The normal stress of the shear test is 10%, 20%, 30%, and 40% of the uniaxial compressive strength of the sample with the corresponding RBP. In order to clearly record the failure process of the specimen, the camera and fill light are used. In the present study, all specimen preparation studies were carried out in accordance with the procedures of ASTM D5607 (2014) [29] in the laboratory.

The failure characteristics of the sample are mainly captured from the video captured by the camera. When the sample is ready to be destroyed, the video is selected frame by frame to select the typical failure characteristics.

Through the uniaxial compression test and the direct shear test on the pure matrix sample, the strength parameters of the matrix and rock block are shown in Table 1. It can be found that the Uniaxial compressive strength (UCS), shear stress, cohesion, and internal friction angle of the block are smaller than those of the samples with a strong matrix and larger than those with a weak matrix.

## 3. Results and Analysis

### 3.1. Uniaxial Compression Strength and Failure Characteristics

**Figure 3:**
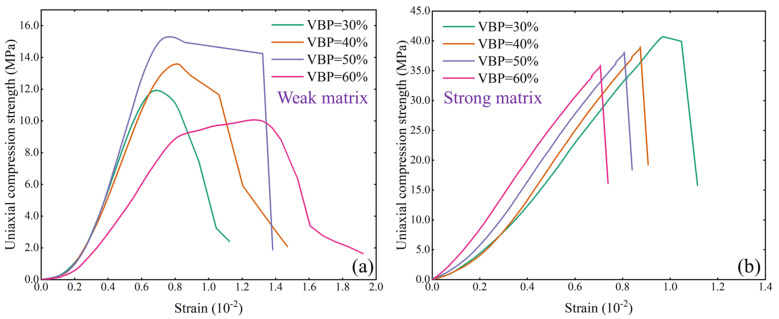
Stress-strain curve. (**a**) The bimrocks with weak matrix and (**b**) the bimrocks with strong matrix.

The stress–strain curves of bimrock specimens with different block-to-matrix strength ratios are quite different. When the strength of the matrix is less than the strength of the block (Figure 3a), the elastic modulus of the sample is similar as the VBP increases from 30% to 50%, and the uniaxial compressive strength of the sample increases with the increase in the VBP. However, the elastic modulus and uniaxial compressive strength of the sample decrease when the VBP increases to 60%, and the curve compaction stage of the sample with a VBP of 60% is obviously longer. In addition, it can also be found that with the increase in VBP, the peak falling speed of the curve slows down and the peak stress can be maintained for a long time when the VBP is at either 50% or 60%. When the strength of the matrix is higher than that of the block (Figure 3b), the elastic modulus of the sample increases with the increase in the VBP, but the uniaxial compressive strength decreases gradually. The post-peak stage is significantly different from that of the weak matrix type. The strength of the strong matrix sample decreases rapidly after reaching the peak, which also indicates that the bimrock’s brittleness of the strong matrix type is significantly higher than that of the weak matrix sample.

**Figure 4:**
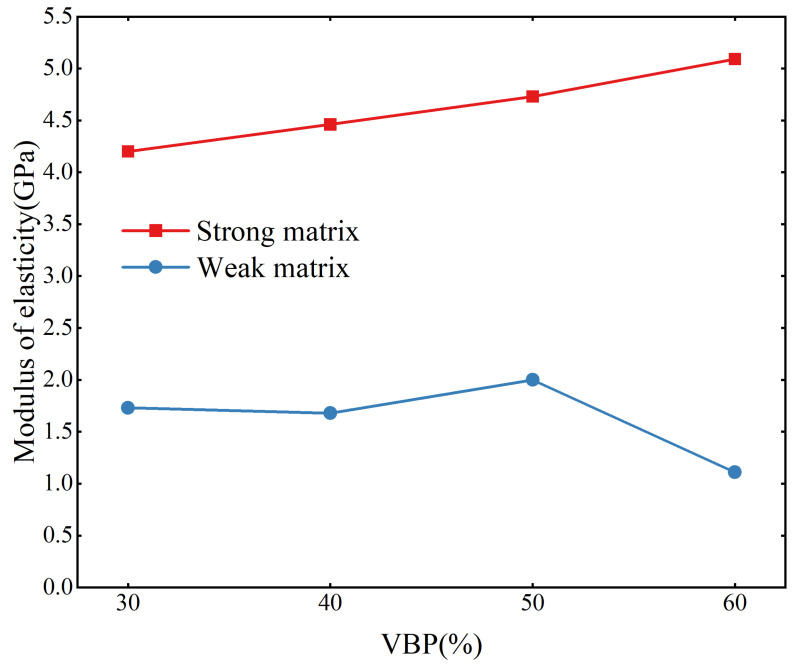
The relationship between VBP and elastic modulus.

The elastic modulus is one of the important indexes for evaluating rock mass strength. Changes in rock mass content in bimrocks will lead to changes in rock mass strength, which is influenced by the block-to-matrix strength ratio. It can be seen from Figure 4 that for the samples with a strong matrix, the elastic modulus of the sample shows a linear increase as the VBP increases from 30% to 60%. However, in the weak matrix samples, there is no obvious change; only in the case of 60% of the high stone content, the elastic modulus is small. These results show that the difference of the block-to-matrix strength ratio will obviously affect the effect of the content of the blocks on the elastic modulus of the sample, and also indirectly affect the strength of the sample.

**Figure 5:**
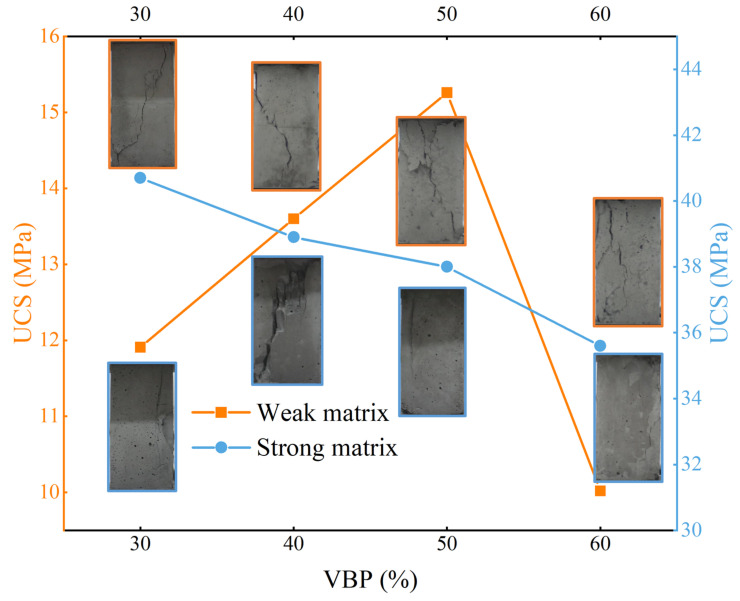
The relationship between VBP and UCS.

In terms of the UCS of the samples, the UCS of weak matrix samples first increases and then decreases with the increase in VBP. When the VBP is 50%, it reaches the maximum value (Figure 5). As the VBP increases to 60%, the UCS of samples decreases greatly, indicating that the matrix content is relatively small at this time, and the blocks are not completely bonded, so the strength decreases. The UCS of the strong matrix sample is significantly higher than that of the weak matrix sample and shows a decreasing trend with the increase in VBP, but the decrease is significantly smaller than the increase in the weak matrix sample, indicating that the influence of the block stone in the strong matrix sample is obviously weakened.

The strength of the matrix can be roughly evaluated from the failure mode of the sample. The test results show that the failure crack penetration of the weak matrix sample is more common, and with the increase in VBP, the crack gradually changes from an inclined distribution to a vertical distribution, and the number of cracks gradually increases. As shown by the stress–strain curve of the strong matrix sample, the failure time of the sample is short, and the ‘rock burst’ failure occurs at the moment when the sample has a small crack. From the recorded damage photos, it can be found that the sample will occur rapidly along the crack when the crack opening is small. Different from the weak matrix bimrocks, with the increase in VBP, the shape of the sample crack gradually changes from a vertical distribution to a bending distribution.

### 3.2. Shear Strength and Behavior

**Figure 6:**
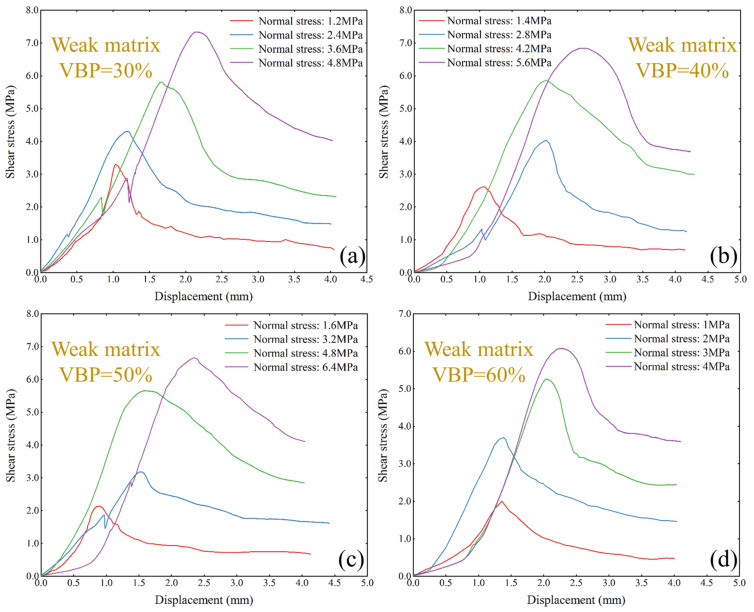
The shear–stress displacement curves of weak matrix bimrocks under different VBPs. (**a**) VBP is 30%, (**b**) VBP is 40%, (**c**) VBP is 50%, and (**d**) VBP is 60%.

The results of the uniaxial compression test show that the UCS of weak matrix bimrocks changes greatly with the increase in VBP, while the UCS of strong matrix bimrocks changes little (Figure 6). Therefore, when the normal stress is set, the normal strength of the weak matrix direct shear specimens with different VBPs is slightly different, and the normal stress of the strong matrix specimens remains the same. In the weak matrix bimrock sample, the peak shear strength of the sample increases with the increase in normal stress, but the increase in amplitude is different under different VBPs. It can be found that after the VBP exceeds 40%, the peak shear strength of the two samples with a relatively large normal stress is similar. When the VBP is 30%, the peak shear strength of the two samples with a relatively small normal stress is similar, indicating that in the case of a low VBP, a small normal stress has little effect on the shear strength of the sample. With the increase in VBP, a large normal stress has little effect on the shear strength of the sample. It can be found that when the VBP is 30%, with the increase in normal stress, the peak shear strain of the sample increases gradually, but with the increase in VBP, the influence of normal stress on the peak shear strain is not obvious.

**Figure 7:**
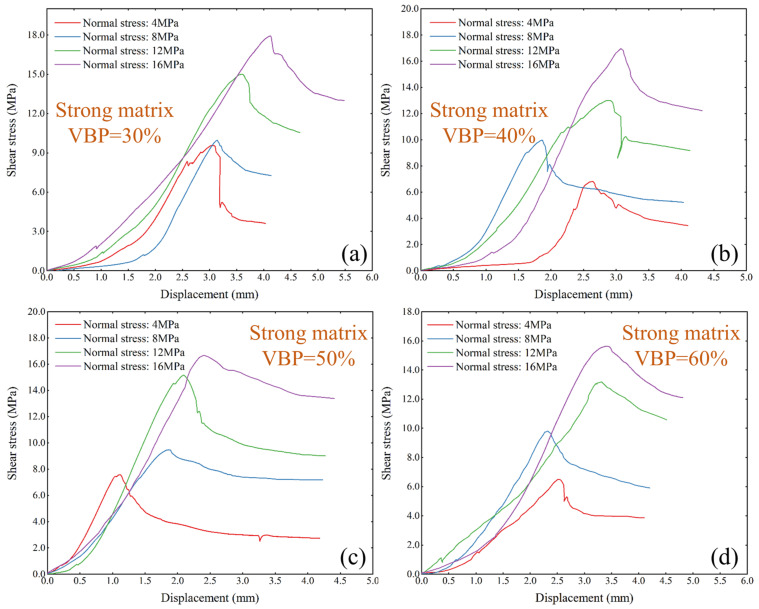
The shear–stress displacement curves of strong matrix bimrocks under different VBPs. (**a**) VBP is 30%, (**b**) VBP is 40%, (**c**) VBP is 50%, and (**d**) VBP is 60%.

In the strong matrix bimrocks (Figure 7), the increase in normal stress from 4 MPa to 8 MPa has little effect on the shear strength of the sample when the VBP is 30%. With the increase in VBP, the difference of shear strength between the two is gradually obvious. As the VBP exceeds 50%, the shear strength of the sample is small, when the normal stress is 12 MPa to 16 MPa, it is similar to that of the weak matrix bimrocks. The difference is that the threshold of the VBP increases. When the VBP is 30%, the peak shear strain of the sample is basically the same under a relatively low normal stress. As the normal stress increases, the peak shear strain increases. When the VBP is 60%, the change of normal stress within a certain range has little effect on the peak shear strain of the sample. For example, when the normal stress increases from 4 MPa to 8 MPa and from 12 MPa to 16 MPa, the peak shear strain of the sample is basically the same. With the increase in VBP, the increase in the rate of shear stress of strong matrix bimrocks is different. Under a low VBP, especially when the VBP is less than 40%, the growth rate of shear strength is from slow to fast. The growth rate of shear strength is relatively stable and fast after the VBP increases.

**Figure 8:**
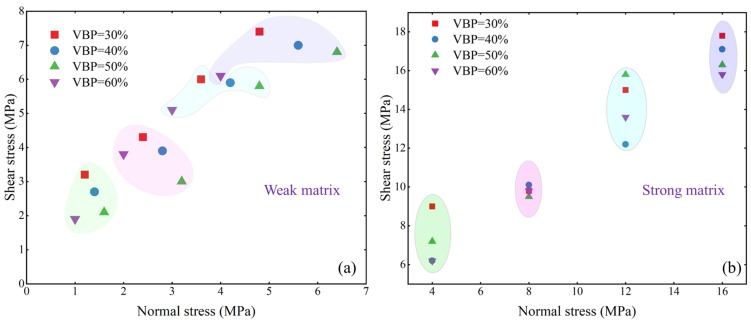
The relationship between normal stress and shear stress. (**a**) Bimrocks with weak matrix, (**b**) bimrocks with strong matrix.

The relationship between the shear strength and normal stress of bimrock specimens with different strength matrices is shown in Figure 7. In the weak matrix sample, because the normal stress of the different VBP samples is slightly different, in order to show the influence of the VBP on the shear strength of the sample under the condition of similar normal stress, the shear strength in the same normal stress range is wrapped with the same color circle (Figure 8a). It can be found that, no matter what the kind of VBP, with the increase in normal stress, the shear strength of the sample shows an increasing trend, but in different normal stress ranges, the influence of the VBP on the shear strength of the sample is different. In the range of normal stress of 2~4 MPa, with the increase in VBP, the shear strength of the sample has no obvious linear relationship. In the other ranges, it shows a decreasing trend and, in the smaller range of normal stress, this decreasing trend is more obvious. In the strong matrix sample (Figure 8b), the variation of the shear strength of the sample with the VBP is different under different normal stresses. It can be clearly found that when the normal stress is 8 MPa, the shear strength of the four VBP samples is relatively similar. There are obvious differences under the different normal stresses, and only in the case of 16 MPa, the shear strength of the sample can decrease with the increase in VBP.

**Figure 9:**
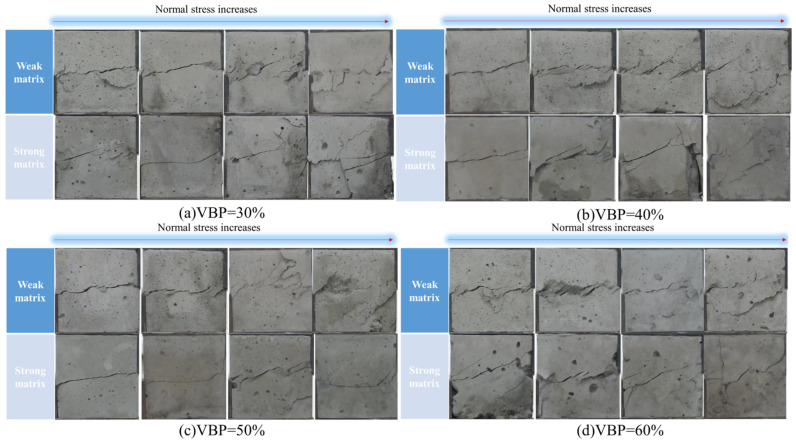
Shear crack distribution of samples under different VBPs.

The specimens with different rock matrix strength ratios are also different in shear failure characteristics (Figure 9). The shear failure surface of the weak matrix specimen has a higher roughness and a larger opening degree, and it is mostly a broken line extension, while the shear fracture of the strong matrix specimen is relatively straight, and the opening degree of the shear failure surface is also small. The normal stress of a sample has a great influence on the shape of the shear failure surface of the sample. It can be found that with the increase in the normal stress, the failure of all the samples is more serious, and the shear zone has a tendency to tilt upward, and the shear failure area of the weak matrix sample is obviously larger than that of the strong matrix sample. The response of different VBP weak matrix specimens to normal stress changes is less different, but the sensitivity of the failure characteristics of the strong matrix specimens to normal stress is affected by the VBP. When the VBP of the specimen is 30%, the failure crack of the specimen with the largest normal stress extends to the bottom of the specimen. With the increase in VBP, the failure crack of the specimen under a large normal stress is mainly distributed along the shear direction and the degree of tortuosity of the crack is increased. The failure characteristics of the specimen with a small normal stress change significantly when the VBP is 60%, the shear zone area becomes larger, and several secondary cracks appear.

### 3.3. Cohesion and Internal Friction Angle

**Figure 10:**
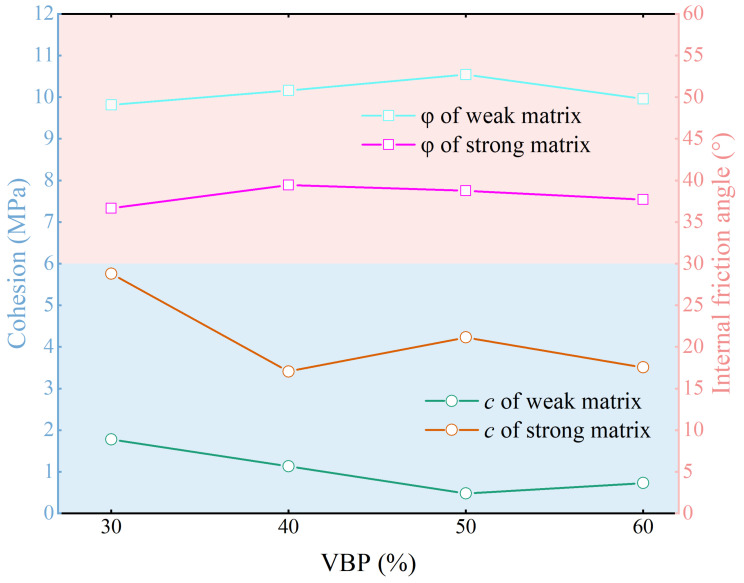
The relationship between internal friction angle and cohesion with VBP.

The cohesion and internal friction angle of the specimens with different matrix rock strength ratios have similar trends with the increase in VBP (Figure 10), and the cohesion shows a decreasing trend. When the VBP of the strong matrix specimen increases from 30% to 40%, the cohesion decreases greatly. As the VBP continues to increase, the cohesion of the specimen fluctuates slightly, and the overall difference is small. The cohesion of the weak matrix specimen increases from 30% to 50%. When the VBP increases to 60%, the cohesion changes little, and the cohesion of the strong matrix specimen is significantly higher than that of the weak matrix specimen. The internal friction angle of the weak matrix sample is obviously higher than that of the strong matrix sample, but with the increase in VBP, the internal friction angle changes little, which corresponds to the cohesion. The internal friction angle of the strong matrix sample changes the most when the VBP increases from 30% to 40%. The results also reveal that the strength ratio of matrix block stone mainly affects the cohesion and internal friction angle, but the influence of the VBP on the internal friction angle is not related to the strength ratio of matrix block stone, but has a certain relationship with the cohesion, especially when the VBP is less than 40%.

## 4. Discussion

Through the relevant mechanical experiments on the indoor-made bimrock-like materials, it is found that the strength ratio of the matrix block stone plays an important role in the influence of the VBP on the strength of the sample.

**Figure 11:**
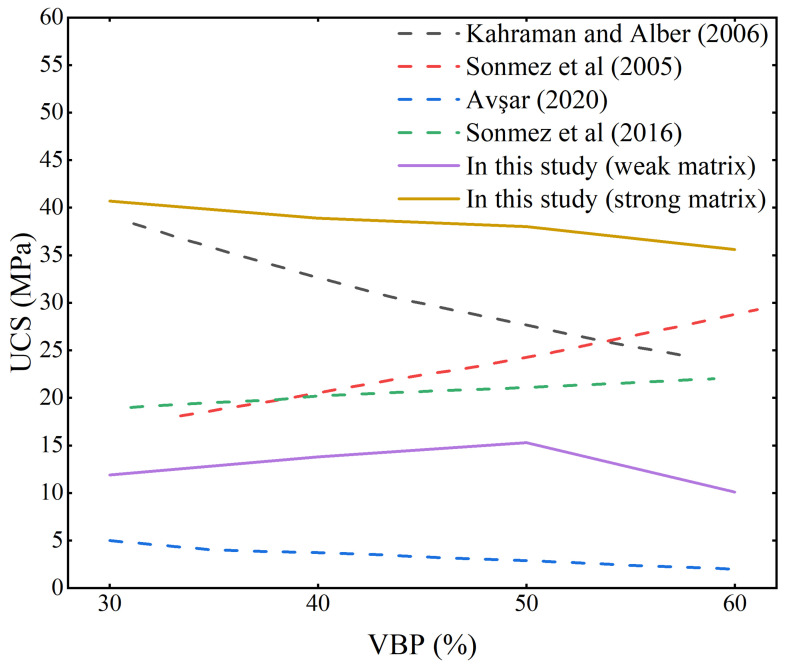
Comparison of UCS changes with VBP.

When the strength of the matrix is weaker than the strength of the block stone, with the increase in the stone content, the UCS of the sample shows a trend of first increasing and then decreasing, which is similar to the research results of Sonmez et al. [25,26], but not exactly the same. Their research results show that when the VBP increases from 30% to 60%, the UCS of the sample mainly shows an increasing trend. The difference in the analysis results is mainly due to the block-to-matrix strength ratio. In the study of Sonmez et al. [25,26], the strength of the block stone is much larger than that of the matrix, so the UCS continues to increase with the increase in VBP. However, in this experiment, the strength of the block stone does not reach a condition that is much higher than that of the matrix. In the case of a high VBP (60%), the phenomenon of interlocking friction between the blocks is more common and there are fewer matrices that play a bonding role, which are more likely to have a low UCS. This result also reveals that when the strength of the block compared with the matrix strength does not exceed a certain value, the strength of the sample with a high stone content, especially the UCS, is not very high due to the low cohesion of the matrix content. The results of the strong matrix samples are the same as those of Kahraman and Alber [28] and Avşar [30], and the UCS of the samples shows a decreasing trend with the increase in VBP (Figure 11), indicating that the definition of strong matrix weak rock type bimrocks has been satisfied under the strength ratio of this matrix rock.

Sheikhpourkhani et al. [31] studied unwelded bimrocks by means of indoor tests and numerical simulation and showed the change rule of the ratio of the sample UCS to the matrix UCS and found that with the increase in VBP, the value showed a decreasing trend, which was similar to the result of bimrocks with a strong matrix. In the following research, the mechanical properties of unwelded bimrocks should also be taken into account.

## 5. Conclusions

New experimental data and the relationship between the matrix and block strength for studying the mechanical properties of bimrock specimens was proposed. Through uniaxial compression tests and direct shear tests on artificial bimrocks, the effects of the VBP on the mechanical behavior of bimrock specimens with different matrix rock strength ratios were obtained. The specific conclusions are as follows:

(1) The stress–strain curve of the weak matrix sample decreases slowly after reaching the peak value. With the increase in VBP, this phenomenon is more obvious. The curve of the strong matrix sample will drop sharply after reaching its peak strength. The VBP is negatively correlated with the peak stress and peak strain of the sample.
(2) With the increase in VBP, the UCS of the strong matrix samples decreases gradually, and the UCS of the weak matrix samples shows an increasing trend as a whole. However, when the VBP is larger than 60%, the strength is lower. The crack opening of weak matrix samples is obviously larger than that of strong matrix samples. With the increase in VBP, the crack distribution of weak matrix samples gradually changes from an inclined distribution to a vertical distribution, while the crack distribution of strong matrix samples is just the opposite.
(3) In the weak matrix sample, the shear strength of the sample decreases with the increase in VBP under various normal stress ranges. However, the influence of VBP in the strong matrix sample is more chaotic. With the increase in normal stress, the failure cracks of weak matrix specimens tend to tilt in the vertical direction, while the secondary cracks extending to the top (or bottom) surface appear in the strong matrix specimens. With the increase in VBP, the shear band of weak matrix specimens increases obviously, while multiple secondary cracks appear in the strong matrix specimens.
(4) The effect of the VBP on cohesion and internal friction angle is less related to the block-to-matrix strength ratio. The internal friction angle shows a slight increasing trend, while the cohesion shows a decreasing trend. The variation of internal friction angle and cohesion of strong matrix samples is relatively large when the VBP is small.

The analysis on the micro level is our next research direction, and this part will be discussed in detail by means of numerical simulation.

## Figures and Tables

**Table 1:** Strength parameters of matrix and rock block.

	UCS (MPa)	Shear Stress (MPa)	Cohesion (MPa)	Internal Friction Angle (°)
Weak matrix	14.2	8.2	3.4	44.3
Strong matrix	41.2	23.6	7.6	36.8
Rock block	32.7	18.7	5.9	38.5

## Data Availability

The data presented in this study are available on request from the corresponding author. The data are not publicly available due to privacy.
